# Supplementary material for: An atomic-resolution view of neofunctionalization in the evolution of apicomplexan lactate dehydrogenases
Source: eLife. 2014 Jun 25;3:e02304. doi: 10.7554/eLife.02304 (PMC4109310; doi:10.7554/eLife.02304)
Supplement: Figure 2—figure supplement 2—source data 1. — DOI: http://dx.doi.org/10.7554/eLife.02304.008 [file elife02304s001.pdf]

	k_{cat} (sec^{-1})	K_M (μM)	$K_{i\ Pyruvate}$ (mM)	k_{cat}/K_M (sec^{-1} M^{-1})
Wild Type	110 ± 2	67 ± 5	59 ± 12	1.6 ± 0.1 ×10^{6}
T101A	39 ± 1	30 ± 4	19 ± 3	1.3 ± 0.2 ×10^{6}
K102A	79 ± 2	460 ± 57	-	1.7 ± 0.2 ×10^{5}
A103S	130 ± 3	180 ± 17	500 ± 890	6.9 ± 0.6 ×10^{5}
P105A	77 ± 2	190 ± 25	-	4.1 ± 0.6 ×10^{5}
G106A	150 ± 4	360 ± 34	130 ± 75	4.2 ± 0.3 ×10^{5}
K107aA	140 ± 3	440 ± 43	-	3.2 ± 0.3 ×10^{5}
S107bA	110 ± 5	100 ± 16	-	1.1 ± 0.2 ×10^{6}
D107cA	98 ± 4	120 ± 18	35 ± 10	8.4 ± 1.2 ×10^{5}
K107dA	74 ± 2	52 ± 6	76 ± 29	1.4 ± 0.2 ×10^{6}
E107eA	190 ± 7	180 ± 24	560 ± 180	1.0 ± 0.1 ×10^{6}
W107fA	0.071 ± 0.002	3000 ± 320	-	2.3 ± 0.2 ×10^{1}
N108A	150 ± 4	550 ± 64	-	2.7 ± 0.3 ×10^{5}
