# Supplementary material for: An atomic-resolution view of neofunctionalization in the evolution of apicomplexan lactate dehydrogenases
Source: eLife. 2014 Jun 25;3:e02304. doi: 10.7554/eLife.02304 (PMC4109310; doi:10.7554/eLife.02304)
Supplement: Figure 4—source data 1. — DOI: http://dx.doi.org/10.7554/eLife.02304.015 [file elife02304s002.pdf]

	Oxaloacetate				Pyruvate			
	k_{cat} (sec^{-1})	K_M (μM)	K_i (mM)	k_{cat}/K_M (sec^{-1} M^{-1})	k_{cat} (sec^{-1})	K_M (μM)	K_i (mM)	k_{cat}/K_M (sec^{-1} M^{-1})
*Pf*MDH	64 ± 4	120 ± 30	14 ± 3.6	5.6 ± 1.2 $\times10^5$	-	-	-	-
*Cp*MDH	570 ± 30	180 ± 20	6 ± 1.1	3.1 ± 0.2 $\times10^6$	-	-	-	-
*Pf*MDH-R102K	0.45 ± 0.04	12000 ± 2000	-	3.7 ± 0.3 $\times10^1$	-	-	-	-
*Pf*MDH-INS	11 ± 1	3300 ± 1600	-	3.2 ± 1.2 $\times10^3$	0.17 ± 0.023	48000 ± 11000	-	3.5 ± 0.4 $\times10^0$
*Cp*MDH-INS	170 ± 10	12100 ± 1850	-	1.6 ± 0.2 $\times10^4$	0.9 ± 0.2	212000 ± 47000	-	4.1 ± 0.2 $\times10^0$
*Pf*MDH-R102K-INS	0.017 ± .001	200 ± 61	-	8.8 ± 2.8 $\times10^1$	0.15 ± 0.01	49000 ± 7400	-	3.1 ± 0.2 $\times10^0$
*Pf*LDH-K102R-DEL	6.3 ± 0.2	2100 ± 250	-	3.1 ± 0.3 $\times10^3$	0.02 ± 0.0007	3000 ± 280	-	6.8 ± 0.5 $\times10^0$
*Pf*LDH-DEL	0.0064 ± 0.0002	2100 ± 310	-	3.1 ± 0.5 $\times10^0$	0.021 ± 0.001	1300 ± 250	-	1.6 ± 0.3 $\times10^1$
*Tg*LDH2-DEL	5.4 ± 2.5	135000 ± 77000	-	5.5 ± 0.4 $\times10^1$	-	-	-	-
*Pf*LDH-K102R	0.7 ± 0.06	12900 ± 2100	-	5.3 ± 0.4 $\times10^1$	150 ± 3	210 ± 14	250 ± 96	7.1 ± 0.4 $\times10^5$
*Pf*LDH	0.17 ± 0.08	139000 ± 93000	-	0.09 ± 0.08 $\times10^0$	110 ± 2	67 ± 5	59 ± 12	1.6 ± 0.1 $\times10^6$
*Tg*LDH2	0.4 ± 0.03	10700 ± 2600	-	4.2 ± 0.9 $\times10^1$	16 ± 0.4	260 ± 35	-	6.0 ± 0.7 $\times10^4$
