# Supplementary material for: An atomic-resolution view of neofunctionalization in the evolution of apicomplexan lactate dehydrogenases
Source: eLife. 2014 Jun 25;3:e02304. doi: 10.7554/eLife.02304 (PMC4109310; doi:10.7554/eLife.02304)
Supplement: Figure 5—source data 1. — DOI: http://dx.doi.org/10.7554/eLife.02304.017 [file elife02304s003.pdf]

	Oxaloacetate				Pyruvate			
	k_{cat} (sec^{-1})	K_M (μM)	K_i (mM)	k_{cat}/K_M (sec^{-1} M^{-1})	k_{cat} (sec^{-1})	K_M (μM)	K_i (mM)	k_{cat}/K_M (sec^{-1} M^{-1})
TgLDH1	1 ± 0.1	26000 ± 2300	-	3.7 ± 0.1 $\times 10^1$	73 ± 2	180 ± 10	47 ± 10	4.2 ± 0.2 $\times 10^5$
RbMDH	250 ± 10	24 ± 2.5	0.84 ± 0.09	1.1 ± 0.07 $\times 10^7$	-	-	-	-
AncLDH	0.03 ± 0.001	2300 ± 400	-	1.3 ± 0.2 $\times 10^1$	8.8 ± 0.4	2.9 ± 0.5	5.9 ± 0.9	3.1 ± 0.6 $\times 10^6$
AncMDH3	10 ± 0.4	1.4 ± 0.4	3.1 ± 0.6	6.9 ± 1.6 $\times 10^6$	0.03 ± 0.001	14800 ± 1000	-	2.0 ± 0.1 $\times 10^0$
AncMDH2	7.5 ± 1	0.4 ± 0.5	0.14 ± 0.06	3.3 ± 6.6 $\times 10^7$	0.07 ± 0.006	22000 ± 4400	-	3.2 ± 0.4 $\times 10^0$
AncMDH1	40 ± 3	3.1 ± 0.6	0.05 ± 0.01	1.2 ± 0.2 $\times 10^7$	0.04 ± 0.001	27800 ± 2400	-	1.3 ± 0.06 $\times 10^0$
