# Supplementary material for: An atomic-resolution view of neofunctionalization in the evolution of apicomplexan lactate dehydrogenases
Source: eLife. 2014 Jun 25;3:e02304. doi: 10.7554/eLife.02304 (PMC4109310; doi:10.7554/eLife.02304)
Supplement: Figure 6—source data 1. — DOI: http://dx.doi.org/10.7554/eLife.02304.020 [file elife02304s004.pdf]

	Oxaloacetate				Pyruvate			
	k_{cat} (sec^{-1})	K_M (μM)	K_i (mM)	k_{cat}/K_M (sec^{-1} M^{-1})	k_{cat} (sec^{-1})	K_M (μM)	K_i (mM)	k_{cat}/K_M (sec^{-1} M^{-1})
AncMDH2*	14 ± 0.8	0.82 ± 0.32	0.35 ± 0.07	1.8 ± 0.6 $\times 10^7$	0.03 ± 0.001	16000 ± 2300	-	1.9 ± 0.2 $\times 10^0$
AncMDH2*-INS	180 ± 20	83 ± 12	0.15 ± 0.02	2.2 ± 0.1 $\times 10^6$	74 ± 5	4000 ± 380	5.2 ± 0.6	1.9 ± 0.1 $\times 10^4$
AncMDH2*-58Mut-R102K	14 ± 0.3	3000 ± 300	-	4.8 ± 0.4 $\times 10^3$	0.03 ± 0.002	5300 ± 750	144 ± 59	6.5 ± 0.5 $\times 10^0$
AncLDH*	1.4 ± 0.1	31000 ± 7800	-	4.5 ± 0.7 $\times 10^1$	63 ± 4	94 ± 23	20 ± 6.5	6.7 ± 1.6 $\times 10^5$
