# Supplementary material for: An atomic-resolution view of neofunctionalization in the evolution of apicomplexan lactate dehydrogenases
Source: eLife. 2014 Jun 25;3:e02304. doi: 10.7554/eLife.02304 (PMC4109310; doi:10.7554/eLife.02304)
Supplement: Figure 6—source data 2. — DOI: http://dx.doi.org/10.7554/eLife.02304.021 [file elife02304s005.pdf]

	Oxaloacetate				Pyruvate			
	**k_{cat} (sec^{-1})**	**K_M (μM)**	**K_i (mM)**	**k_{cat}/K_M (sec^{-1} M^{-1})**	**k_{cat} (sec^{-1})**	**K_M (μM)**	**K_i (mM)**	**k_{cat}/K_M (sec^{-1} M^{-1})**
AncMDH2-59Mut	2.8 ± 0.2	1 ± 0.5	0.58 ± 0.18	2.9 ± 1.4 $\times 10^{6}$	0.12 ± 0.02	2000 ± 1000	-	6.1 ± 2.4 $\times 10^{1}$
AncMDH2-R102K	48 ± 5	570 ± 120	3.7 ± 0.8	8.5 ± 1.1 $\times 10^{4}$	0.047 ± 0.013	16000 ± 10000	-	2.9 ± 1.1 $\times 10^{0}$
AncMDH2-R102K-59Mut	20 ± 1	560 ± 78	15 ± 2.4	3.5 ± 0.4 $\times 10^{4}$	0.066 ± 0.003	2300 ± 530	-	2.8 ± 0.6 $\times 10^{1}$
AncMDH2-INS	52 ± 4.8	27 ± 5	0.23 ± 0.08	2.0 ± 0.4 $\times 10^{6}$	35 ± 2	950 ± 120	4.2 ± 0.5	3.9 ± 0.2 $\times 10^{4}$
AncMDH2-INS-59Mut	26 ± 1.3	48 ± 5	0.048 ± 0.005	5.4 ± 0.6 $\times 10^{5}$	12 ± 1	22 ± 4.2	6.7 ± 1.3	5.7 ± 1.1 $\times 10^{5}$
AncMDH2-R102K-INS	0.25 ± 0.03	350 ± 110	9.1 ± 3.4	7.3 ± 1.4 $\times 10^{2}$	27 ± 1	130 ± 10	3.4 ± 0.2	2.1 ± 0.2 $\times 10^{5}$
