# Supplementary material for: An atomic-resolution view of neofunctionalization in the evolution of apicomplexan lactate dehydrogenases
Source: eLife. 2014 Jun 25;3:e02304. doi: 10.7554/eLife.02304 (PMC4109310; doi:10.7554/eLife.02304)
Supplement: Figure 8—source data 1. — DOI: http://dx.doi.org/10.7554/eLife.02304.032 [file elife02304s006.pdf]

	Oxaloacetate				Pyruvate			
	k_{cat} (sec^{-1})	K_M (μM)	K_i (mM)	k_{cat}/K_M (sec^{-1} M^{-1})	k_{cat} (sec^{-1})	K_M (μM)	K_i (mM)	k_{cat}/K_M (sec^{-1} M^{-1})
AncMDH2-R102L	10 ± 1	2000 ± 320	12 ± 2.3	4.9 ± 0.5 $\times10^3$	2.4 ± 0.1	22000 ± 1300	-	1.1 ± 0.1 $\times10^2$
AncMDH2-R102G	44 ± 10	4300 ± 1300	5 ± 1.5	1.0 ± 0.1 $\times10^4$	6.9 ± 0.1	16000 ± 900	-	4.4 ± 0.2 $\times10^2$
AncMDH2-R102Q	12 ± 0.2	880 ± 40	0.88 ± 0.04	1.3 ± 0.1 $\times10^4$	40 ± 4.2	4500 ± 700	2.9 ± 0.4	9.2 ± 0.4 $\times10^3$
AncMDH2-R102W	0.18 ± 0.008	23000 ± 2400	-	8.1 ± 0.6 $\times10^0$	0.15 ± 0.003	23000 ± 1000	-	6.5 ± 0.2 $\times10^0$
