# Supplementary figures and images for: An atomic-resolution view of neofunctionalization in the evolution of apicomplexan lactate dehydrogenases
Source: eLife. 2014 Jun 25;3:e02304. doi: 10.7554/eLife.02304 (PMC4109310; doi:10.7554/eLife.02304)

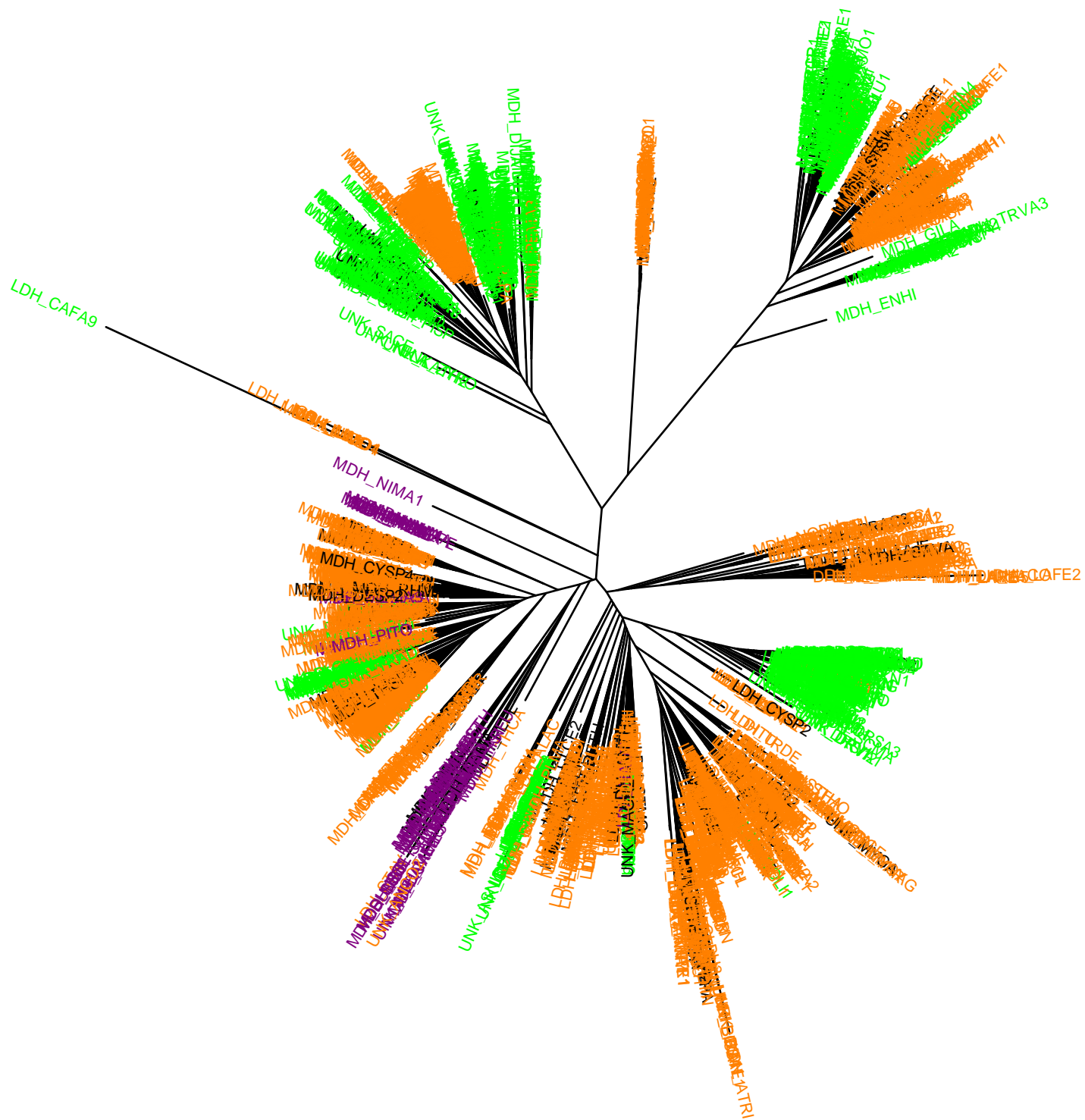

Supplement: Supplementary file 1. — Sequences, alignments, and trees. Alignments and tree files for both the original (Figure 3) and the alternative phylogeny. Alignment for Figure 5—figure supplement 1. Ancestral FASTA files and posterior probabilities for each ancestral sequence (parsed in Figure 6—figure supplements 1–6). DOI: http://dx.doi.org/10.7554/eLife.02304.033 [file elife02304s007.zip › Bioinformatics_Files/Original_LDHMDH_Phylogeny_DomainColored.pdf]
