# Supplementary material for: An atomic-resolution view of neofunctionalization in the evolution of apicomplexan lactate dehydrogenases
Source: eLife. 2014 Jun 25;3:e02304. doi: 10.7554/eLife.02304 (PMC4109310; doi:10.7554/eLife.02304)
Supplement: Supplementary file 2. — Molecular weights and extinction coefficients. ExPASy calculated molecular weights and extinction coefficients for all proteins used within this study. DOI: http://dx.doi.org/10.7554/eLife.02304.034 [file elife02304s008.pdf]

Construct	MW (Da)	ε_{280} (M^{-1} cm^{-1})	Construct	MW (Da)	ε_{280} (M^{-1} cm^{-1})	Construct	MW (Da)	ε_{280} (M^{-1} cm^{-1})
*Pf*LDH	34930.6	17420	*Pf*MDH-R102K	35077.7	11920	AncMDH2-R102K-INS	36382.9	25900
*Pf*LDH_T101A	34900.5	17420	*Pf*MDH-INS	35751.3	17420	AncLDH	36969.6	27390
*Pf*LDH_K102A	34873.5	17420	*Cp*MDH-INS	35335.7	20400	AncMDH2-R102L	35725.3	20400
*Pf*LDH_A103S	34946.6	17420	*Pf*MDH-R102K-INS	35723.3	17420	AncMDH2-R102G	35669.2	20400
*Pf*LDH_P105A	34904.5	17420	*Pf*LDH-K102R-DEL	34312.9	11920	AncMDH2-R102Q	35740.3	20400
*Pf*LDH_G106A	34944.6	17420	*Pf*LDH-DEL	34284.9	11920	AncMDH2-R102W	35798.3	25900
*Pf*LDH_K107aA	34873.5	17420	*Tg*LDH2-DEL	35729.5	11920	AncMDH2*	35360.1	11920
*Pf*LDH_S107bA	34914.6	17420	*Pf*LDH-K102R	34958.6	17420	AncMDH2*-INS	36002.8	17420
*Pf*LDH_D107cA	34886.5	17420	*Tg*LDH2	36372.2	17420	AncMDH2*-58Mut-R102K	35964.8	11920
*Pf*LDH_K107dA	34873.5	17420	AncMDH2	35768.3	20400	AncLDH*	36607.4	17420
*Pf*LDH_E107eA	34872.5	17420	AncMDH2-59Mut	36355.0	21890	AncMDH3	35285.0	11920
*Pf*LDH_W107fA	34815.4	11920	AncMDH2-R102K	35740.3	20400	AncMDH1	35188.6	21890
*Pf*LDH_N108A	34887.5	17420	AncMDH2-R102K-59Mut	36327.0	21890	*Tg*LDH1	36613.5	23380
*Pf*MDH	35105.7	11920	AncMDH2-INS	36411.0	25900	*Rb*MDH	34716.3	14900
*Cp*MDH	34693.1	14900	AncMDH2-INS-59Mut	36997.6	27390			
